# Specialist prescribing of psychotropic drugs to older persons in Sweden - a register-based study of 188 024 older persons

**DOI:** 10.1186/1471-244X-12-197

**Published:** 2012-11-13

**Authors:** Gunilla Martinsson, Ingegerd Fagerberg, Lena Wiklund-Gustin, Christina Lindholm

**Affiliations:** 1 Department of Neurobiology, Care Sciences and Society, Karolinska Institutet, Stockholm, Sweden; 2 School of Health, Care and Social Welfare, Mälardalen University, Västerås, Sweden; 3 Department of Health Care Sciences, Ersta Sköndal University College, Stockholm, Sweden; 4 Faculty of Health and Society, Narvik University College, Narvik, Norway; 5 Department of Clinical Neuroscience, Karolinska Institutet, Stockholm, Sweden

**Keywords:** Aged, Geriatric, Mental disorders, Older persons, Physicians, Prescribing, Psychiatry, Psychotropic drugs, Register-based

## Abstract

**Background:**

The situation for older persons with mental disorders other than dementia disorders has scarcely been studied. The older population is increasing worldwide and along with this increase the prevalence of mental disorders will also rise. The treatment of older persons with mental disorders entails complex challenges, with drugs constituting the major medical treatment. Knowledge of geriatric psychiatry is essential for providing older persons with appropriate treatment and care. This study aimed to evaluate the prescription of drugs for mental disorders to older persons (≥65) in Sweden, focused on the medical specialties of the prescribing physicians.

**Methods:**

Data concerning drug treatment for older persons from 2006 to 2008 was gathered from the Swedish Prescribed Drug Register. Mental disorders, defined as affective, psychotic and anxiety disorders (ICD-10 F20-42) were evaluated in order to identify associated drugs. Included was a total of 188 024 older individuals, who collectively filled 2 013 079 prescriptions for the treatment of mental disorders. Descriptive analyses were performed, including frequency distribution and 95% CI. The competence of the prescribers was analyzed by subdividing them into five groups: geriatricians, psychiatrists, general practitioners (GPs), other specialists, and physicians without specialist education.

**Results:**

GPs represented the main prescribers, whereas geriatricians and psychiatrists rarely prescribed drugs to older persons. Benzodiazepines and tricyclic antidepressants were the most commonly prescribed drugs. Women were prescribed drugs from geriatricians and psychiatrists to a greater extent than men.

**Conclusions:**

This study examined the prescription of psychotropic drugs to older persons. Physicians specialized in older persons’ disorders and mental health were rarely the prescribers of these drugs. Contrary to clinical guidelines, benzodiazepines and tricyclic antidepressants were commonly prescribed to older persons, emphasizing the need for continuous examination of pharmaceutical treatment for older persons. The results indicate a future need of more specialists in geriatrics and psychiatry.

## Background

The world is facing a demographical shift in which the older population will increase. It is thus fair to assume that the prevalence of disorders that are common among older persons will also increase. Studies have shown that mental disorders are prevalent among the old [1-3], even when dementia disorders are excluded [4,5]. The treatment of mental disorders (i.e. affective, psychotic and anxiety disorders) in older persons is a complex task and can be affected by a variety of factors, including pharmacokinetic and pharmacodynamic changes related to age, influences of concurrent disorders and possible adverse drug reactions. Although the risk of adverse drug reactions increases with age, drug treatment is common among older persons, both nationally [6] and internationally [7]. As older persons are often treated for several conditions besides mental disorders, the possibility for unwanted drug interactions is increased in this age group. Indeed, several studies have reported that older persons are subjected to polypharmacy and inappropriate drug use [8-10], and that many hospitalizations occur due to drug related problems [11].

The treatment of mental disorders often involves several different physicians, raising questions about who ultimately carries the responsibility for the adequacy and quality of the combined pharmaceutical regime. In addition, the time allocated to mental health issues during consultations with physicians is generally low [12] and the combination of several prescribers commonly entails more drugs and lower overall quality of care [13]. At present, very little time (at most two weeks) is allocated to the field of geriatrics during the training to become a licensed physician in Sweden [14]. Specialists in geriatrics and psychiatry represent approximately 2% and 6%, respectively, of the licensed physicians in Sweden, while physicians with specialist education in general medicine (general practitioners, GPs) represent 18% [15]. Hence, there is an obvious lack of physicians with specialized skills in treating older persons with mental disorders.

GPs providing care for older persons have reported feeling insecure when required to surpass their own competence, as well as a lack of communication with other physicians and specialists [16]; these factors can negatively impact the treatment and care given to older persons with mental disorders. Studies have demonstrated that treatment by geriatric teams reduces mortality [17,18] and increases the possibility for older persons to remain at home [19,20]. Although the particular knowledge that geriatricians acquire is vital in providing older persons with adequate treatment and care, health care personnel tend to prefer interdisciplinary teams comprising different medical specialties when treating and caring for older persons with or without mental disorders [21,22]. Psychiatrists in particular contribute important knowledge on mental health and, as they generally hold more positive professional attitudes towards people with mental disorders such as schizophrenia [23], they would preferably be included in such a team.

The current situation for older persons with mental disorders has scarcely been studied and questions have been raised as to whether or not older persons with extensive needs receive adequate care and treatment. In order to facilitate the improvement of treatment and to reduce possible adverse drug reactions among older persons with mental disorders, it is important to evaluate the amounts and types of drugs older persons are being prescribed and which medical specialties their prescribers hold.

### Aim

This study aimed to evaluate the prescription of drugs for mental disorders to older persons (≥65 years) in Sweden during the three-year period from 2006 to 2008, focusing on the medical specialties among the prescribing physicians and the amount of drugs prescribed to older men and women.

## Methods

### Study population

All persons in Sweden aged 65 years and older were included in this analysis if they had received one or more registered prescriptions for drugs for mental disorders included in the International Classification of Diseases, version 10 (ICD-10) F20-F42 [24] during 2006, 2007 or 2008. A total of 188 024 individuals, who together received 2 013 079 dispensed drugs for mental disorders during the years 2006, 2007 and 2008, were included in the study.

### Data measurement

The Swedish Prescribed Drug Register (SPDR) from the Swedish National Board of Health and Welfare was the source for the data included in the analysis. All personal information was replaced with serial numbers. The variables included were age (65 years and older), sex, ATC codes (level 5; chemical substance), prescribing and dispensing dates, as well as the specialist codes for the prescribers.

### Data collection and analysis

The SPDR was used to explore the prescription of drugs for mental disorders to older persons. Complete information about the drugs dispensed at national pharmacies is included in the register. As social security numbers were introduced to the register in 2005, the analyses were carried out using data from 2006 to 2008. Older persons receiving prescriptions for drugs indicated or recommended in the Swedish Medicines Information portal [25-28] for use in psychotic (F20-29, ICD-10), affective (F30-39, ICD-10) and anxiety (F41-42, ICD-10) disorders, Chapter V, ICD-10 [24] were included in the analysis. The Anatomical Therapeutic Chemical Classification (ATC) codes, level 5, were subsequently applied (see Table 1). Drugs intended for use in dementia disorders (ICD-10: F00-03; ATC: N06DA02-04, N06DX01) or recommended as firsthand choice in dementia disorders (ATC: N05AA-AX, N05CF-CM, N06AB, N06DA-DX) have been excluded to avoid analyzing persons with diffuse symptoms that could be classified as either mental disorders or dementia disorders. All prescriptions filled and dispensed during the three-year period were included in the analysis.

**Table 1:** Evaluated disorders from International Classification of Diseases version 10 (ICD-10), Chapter V Mental and behavioral disorders: F20-29 (schizophrenia, schizotypal and delusional disorders), F30-39 (affective disorders), F41 (other anxiety disorders) and F42 (obsessive-compulsive disorders); the applied ATC codes level 5 and the corresponding chemical substances included in the study

**ATC level 5**	**Chemical substance**	**F20-29**	**F30-39**	**F41**	**F42**
N03AE01	Clonazepam				X
N03AF01	Carbamazepin		X		
N03AG01	Valproic acid		X		
N03AX09	Lamotrigine		X		
N03AX16	Pregabalin			X	
N04BC05	Pramipexole		X		
N05BA01	Diazepam	X	X	X	
N05BA04	Oxazepam	X	X	X	
N05BA06	Lorazepam	X	X	X	
N05BA12	Alprazolam	X	X	X	
N05BE01	Buspirone		X	X	
N06AA04	Clomipramine	X		X	X
N06AA06	Trimipramine	X	X		
N06AA09	Amitriptyline	X	X		
N06AA10	Nortriptyline	X	X		
N06AA21	Maprotiline	X	X		
N06AG02	Moclobemide		X		
N06AX02	Tryptophan		X		
N06AX03	Mianserin		X		
N06AX11	Mirtazapine		X		
N06AX12	Bupropion		X		
N06AX16	Venlafaxine		X		
N06AX18	Reboxetin		X	X	
N06AX21	Duloxetine		X		
N06BA07	Modafinil		X		

The Swedish system for attaining a specialist degree as a physician includes at least five years of full-time specialization education following undergraduate studies and involves working under supervision and attending complementary education. During the years 2006, 2007 and 2008 there were a total of 62 different specialties possible for physicians, and a given physician may hold several different specialties. In the data analyses, the specialist categories were divided into four groups: general practitioners (GPs), geriatricians, psychiatrists (including specialist education in forensic psychiatry as well as children and young peoples’ psychiatry) and “other specialists” comprising all other specialists. Licensed physicians with no specialist education were analyzed separately. Descriptive univariate analyses with frequency distribution and 95% CI were carried out using IBM SPSS Statistics 19.0 for Windows (SPSS Inc. an IBM company, 1989–2010).

The regional ethics board in Uppsala, Sweden critically and ethically revised and approved this study to be compliant with the Helsinki Declaration [29] (Reference number 2008/345/2).

## Results

On average, 7% of the older population was prescribed drugs for mental disorders during the years 2006, 2007 and 2008 (see Table 2), with more than one-half million prescriptions dispensed to the older population at national pharmacies each year. Women constituted two-thirds and men one-third of the older population receiving these drugs.

**Table 2:** **Characteristics of the study population: number of persons in the older population and number of persons (*n*) receiving dispensed drugs for mental disorders (MD) (ICD-10: F20-F42), mean age with standard deviation (SD), percentage men and women in the study population, and total number of dispensed drugs for mental disorders**

	**2006**	**2007**	**2008**
Older population, total (*n*)	1 581 437	1 608 413	1 645 081
Persons receiving drugs for MD (*n*)	104 441	106 665	108 306
Mean age (years) (SD)	77.8 (7.8)	77.8 (7.8)	77.7 (7.9)
Men/Women (%)	35/65	35/65	36/64
Total number of dispensed drugs for MD	639 841	671 071	702 167

Drugs corresponding to 25 different ATC codes, level 5, were dispensed to older persons (≥65) in Sweden from 2006 to 2008, all of which were included in the analysis (see Table 3). Each ATC code corresponds to one specific chemical substance and may have several brand names. The most commonly prescribed and dispensed chemical substances during each of the three years were the benzodiazepines oxazepam and diazepam (anxiolytics), as well as the non-selective monoamine reuptake inhibitor amitriptyline (antidepressant). The amount and frequency of prescriptions for many of these chemical substances changed over the course of the three years: a decrease was seen in prescriptions for 48% of the substances and an increase for 24%.

**Table 3:** Frequency of ATC codes prescribed to the study population

**ATC**	**Chem. Sub.**	**ICD**	**2006**			**2007**			**2008**		
			***n***	**%**	**95% CI**	***n***	**%**	**95% CI**	***n***	**%**	**95% CI**
N03AE01	Clonazepam	42	1015	0.97	0.91-1.03	1082	1.01	0.95-1.07	1097	1.01	0.95-1.07
N03AF01	Carbamazepin	31	7917	7.58	7.42-7.74	7877	7.38	7.23-7.54	7814	7.21	7.06-7.37
N03AG01	Valproic acid	31	2220	2.13	2.04-2.21	2308	2.16	2.08-2.25	2377	2.19	2.11-2.28
N03AX09	Lamotrigine	31	1259	1.21	1.14-1.27	1436	1.35	1.28-1.42	1691	1.56	1.49-1.64
N03AX16	Pregabalin	41	2641	2.53	2.43-2.62	3982	3.73	3.62-2.85	5231	4.83	4.70-4.96
N04BC05	Pramipexole	30-39	6188	5.92	5.78-6.07	7807	7.32	7.16-7.48	9093	8.40	8.23-8.56
N05BA01	Diazepam	20-39, 41	20075	19.22	18.98-19.46	19315	18.11	17.88-18.34	18477	17.06	16.84-17.28
N05BA04	Oxazepam	20-39, 41	41521	39.76	39.46-40.05	41725	39.12	38.82-39.41	41226	38.06	37.78-38.35
N05BA06	Lorazepam	20-39, 41	1175	1.13	1.06-1.19	1119	1.05	0.99-1.11	1093	1.01	0.95-1.07
N05BA12	Alprazolam	20-39, 41	3992	3.82	3.71-3.94	3910	3.67	3.55-3.78	3819	3.53	3.42-3.64
N05BE01	Buspirone	30-39, 41	473	0.45	0.41-0.49	422	0.40	0.36-0.43	401	0.37	0.33-0.41
N06AA04	Clomipramine	20-29, 41-42	2841	2.72	2.62-2.82	2827	2.65	2.55-2.75	2739	2.53	2.44-2.62
N06AA06	Trimipramine	20-39	342	0.33	0.29-0.36	196	0.18	0.16-0.21	48	0.04	0.03-0.06
N06AA09	Amitriptyline	20-39	11914	11.41	11.21-11.60	11907	11.16	10.97-11.35	12275	11.33	11.14-11.52
N06AA10	Nortriptyline	20-39	381	0.36	0.33-0.40	367	0.34	0.31-0.38	307	0.28	0.25-0.32
N06AA21	Maprotiline	20-39	413	0.40	0.36-0.43	342	0.32	0.29-0.35	322	0.30	0.26-0.33
N06AG02	Moclobemide	30-39	186	0.18	0.15-0.20	167	0.16	0.13-0.18	152	0.14	0.12-0.16
N06AX02	Tryptophan	30-39	36	0.03	0.02-0.05	37	0.03	0.02-0.05	28	0.03	0.02-0.04
N06AX03	Mianserin	30-39	1575	1.51	1.43-1.58	1527	1.43	1.36-1.50	1523	1.41	1.34-1.48
N06AX11	Mirtazapine	30-39	9296	8.90	8.73-9.07	10581	9.92	9.74-10.10	11500	10.62	10.43-10.80
N06AX12	Bupropion	30-39	1552	1.49	1.31-1.56	1264	1.19	1.12-1.25	838	0.77	0.72-0.83
N06AX16	Venlafaxine	30-39	3792	3.63	3.52-3.74	3848	3.61	3.50-3.72	4126	3.81	3.70-3.92
N06AX18	Reboxetin	30-39, 41	91	0.09	0.07-0.11	74	0.07	0.05-0.09	91	0.08	0.07-0.10
N06AX21	Duloxetine	30-39	1480	1.42	1.35-1.49	1602	1.50	1.43-1.57	2036	1.88	1.80-1.96
N06BA07	Modafinil	30-39	95	0.09	0.07-0.11	125	0.12	0.10-0.14	136	0.13	0.10-0.15

ATC codes (ATC), the corresponding chemical substances (Chem. Sub.) and ICD-10 codes F20-42 (ICD) (Chapter V Mental and behavioral disorders) to which the ATC codes can be prescribed, number of older persons (*n*) prescribed each of ATC-codes level 5 and percentage (%) of the older population being prescribed drugs for mental disorders, with 95% CI, during 2006, 2007 and 2008

**Figure 1:**
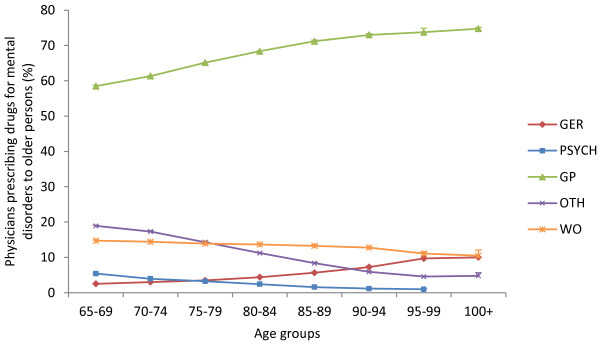
**Percentage of physicians with specialist education in geriatrics (GER), psychiatry (PSYCH), general practitioners (GP), other specialists (OTH) or physicians without specialist education (WO) prescribing drugs for mental disorders (Y-axis) to older persons, in 5-year age brackets (X-axis).** Data are presented as average values for the 3-year period (2006 to 2008) with SEM.

On average, during the period from 2006 to 2008, two-thirds (65%) of the prescribing physicians were GPs (see Figure 1), whereas approximately 4% were geriatricians and 3% psychiatrists. Approximately 14% of the prescribers were physicians with specialist education other than the aforementioned, the majority of whom were specialists in internal medicine, cardiology or neurology. Physicians without any specialist education corresponded to 14% of the prescribers. The tendency of GPs and geriatricians to hand out prescriptions increased with the age of the patient, while the amount of prescriptions from psychiatrists, other specialists, and physicians with no specialist education decreased with age.

Among the dispensed substances, the most commonly prescribed were two benzodiazepine derivatives and one non-selective monoamine reuptake inhibitor (see Table 4). During 2006 to 2008, an average of 39% of the study population was prescribed the anxiolytic oxazepam, while approximately 18% was prescribed diazepam. The antidepressant amitriptyline was prescribed to 11% of the study population yearly, from 2006 to 2008. GPs represented the category of physicians that most commonly prescribed these top three ATC codes. While men and women both received equal amounts of prescriptions from geriatricians and psychiatrists, men received prescriptions from other specialists and physicians with no specialist education to a higher extent than women.

**Table 4:** **The three most commonly prescribed drugs (classification, ATC-code level 5 and chemical substance) for mental disorders (ICD-10) from 2006 to 2008: number (*n*) of and percentage (%), with 95% CI, of older persons (men and women) prescribed drugs for mental disorders by specialists in geriatrics (GER), psychiatry (PSYCH), general practitioners (GP), other specialists (OTH) or by physicians without specialist education (WO)**

**Classification ATC Chem. Sub.**	**ICD-10**	**Specialists**	**Patients’ sex**	**2006**			**2007**			**2008**		
				***n***	**%**	**95% CI**	***n***	**%**	**95% CI**	***n***	**%**	**95% CI**
Benzodiazepine derivative	F20-39, 41	GER	Men	470	4.0	3.6-4.3	492	4.1	3.7-4.4	551	4.6	4.2-4.9
			Women	1218	4.1	3.9-4.3	1302	4.4	4.2-4.6	1378	4.7	4.5-5.0
N05BA04		PSYCH	Men	394	3.3	3.0-3.7	405	3.4	3.0-3.7	413	3.4	3.1-3.8
Oxazepam			Women	815	2.7	2.6-2.9	785	2.6	2.5-2.8	793	2.7	2.5-2.9
		GP	Men	8042	68.3	67.4-69.1	8285	68.6	67.7-69.4	8148	67.7	66.8-68.5
			Women	22132	74.4	73.9-74.9	22099	74.6	74.1-75.0	21655	74.2	73.7-74.7
		OTH	Men	1613	13.7	13.1-14.3	1636	13.5	12.9-14.1	1677	13.9	13.3-14.5
			Women	2929	9.8	9.5-10.2	2905	9.8	9.5-10.1	2816	9.6	9.3-10.0
		WO	Men	1591	13.5	12.9-14.1	1621	13.4	12.8-14.0	1618	13.4	12.8-14.0
			Women	3462	11.6	11.3-12.0	3462	11.7	11.3-12.0	3419	11.7	11.3-12.1
Benzodiazepine derivative	F20-39, 41	GER	Men	262	3.5	3.1-3.9	233	3.2	2.8-3.6	247	3.6	3.2-4.0
			Women	477	3.8	3.4-4.1	485	4.0	3.7-4.4	481	4.1	3.8-4.5
N05BA01		PSYCH	Men	290	3.9	3.5-4.3	295	4.0	3.6-4.5	280	4.1	3.6-4.6
Diazepam			Women	415	3.3	3.0-3.6	411	3.4	3.1-3.7	422	3.6	3.3-4.0
		GP	Men	4820	64.6	63.6-65.7	4799	65.8	64.7-66.9	4501	65.7	64.6-66.8
			Women	9024	71.5	70.7-72.3	8577	71.4	70.6-72.2	8324	71.6	70.8-72.4
		OTH	Men	1374	18.4	17.5-19.3	1263	17.3	16.4-18.2	1145	16.7	15.8-17.6
			Women	1727	13.7	13.1-14.3	1606	13.4	12.8-14.0	1510	13.0	12.4-13.6
		WO	Men	902	12.1	11.4-12.8	886	12.1	11.4-12.9	854	12.5	11.7-13.2
			Women	1308	10.4	9.8-10.9	1280	10.7	10.1-11.2	1239	10.7	10.2-11.2
Non-selective monoamine reuptake inhibitor	F20-39	GER	Men	125	3.6	3.0-4.3	107	3.2	2.6-3.7	106	2.9	2.4-3.5
			Women	269	3.2	2.8-3.5	280	3.3	2.9-3.7	276	3.2	2.8-3.6
		PSYCH	Men	101	2.9	2.4-3.5	95	2.8	2.2-3.4	97	2.7	2.2-3.2
N06AA09			Women	269	3.2	2.8-3.5	251	2.9	2.6-3.3	254	2.9	2.6-3.3
Amitriptyline		GP	Men	2133	62.1	60.5-63.8	2172	64.0	62.4-65.6	2276	63.1	61.5-64.7
			Women	5868	69.2	68.2-70.2	5957	70.0	69.0-70.9	6082	70.2	69.2-71.1
		OTH	Men	585	17.0	15.8-18.3	553	16.3	15.1-17.5	624	17.3	16.1-18.5
			Women	1038	12.2	11.5-12.9	1100	12.9	12.2-13.6	1133	13.1	12.4-13.8
		WO	Men	568	16.5	15.2-17.8	531	15.7	14.4-16.9	569	15.8	14.6-17.0
			Women	1254	14.8	14.0-15.5	1143	13.4	12.7-14.1	1145	13.2	12.5-13.9

Each year approximately 15% of the older population received two or more substances for mental disorders on one or recurrent occasions during the years 2006, 2007 and 2008 (see Table 5). The prescription of one chemical substance tended to decrease, while there was a minor increase in the prescription of two and three chemical substances during the three years.

**Table 5:** **Number (*n*) and percentage (%) of the older population age ≥65 in Sweden receiving prescriptions for one to eight chemical substances for mental disorders, during the years 2006, 2007 and 2008**

**ATC**	**2006**						**2007**						**2008**					
	**Total**		**Men**		**Women**		**Total**		**Men**		**Women**		**Total**		**Men**		**Women**	
	***n***	**(%)**	***n***	**(%)**	***n***	**(%)**	***n***	**(%)**	***n***	**(%)**	***n***	**(%)**	***n***	**(%)**	***n***	**(%)**	***n***	**(%)**
1	89046	(85.3)	31380	(86.5)	57666	(84.6)	90369	(84.7)	32314	(85.8)	58055	(84.1)	91368	(84.4)	32960	(85.6)	58407	(83.7)
2	13191	(12.6)	4175	(11.5)	9015	(13.2)	13871	(13.0)	4550	(12.1)	9320	(13.5)	14308	(13.2)	4695	(12.2)	9613	(13.8)
3	1848	(1.77)	599	(1.65)	1249	(1.83)	2037	(1.91)	662	(1.76)	1375	(1.99)	2160	(1.99)	688	(1.79)	1472	(2.11)
4	295	(0.28)	81	(0.22)	214	(0.31)	328	(0.31)	102	(0.27)	226	(0.33)	387	(0.36)	136	(0.35)	251	(0.36)
5	51	(0.05)	19	(0.05)	32	(0.05)	49	(0.05)	11	(0.03)	38	(0.06)	72	(0.07)	24	(0.06)	48	(0.07)
6	7	(0.01)	3	(0.01)	4	(0.01)	9	(0.01)	4	(0.01)	5	(0.01)	9	(0.01)	6	(0.02)	3	(0.00)
7	2	(0.00)	1	(0.00)	1	(0.00)	2	(0.00)			2	(0.00)	2	(0.00)	2	(0.01)		
8	1	(0.00)	1	(0.00)														
Total	104441	(100)	36259	(100)	68181	(100)	106665	(100)	37643	(100)	69021	(100)	108306	(100)	38511	(100)	69794	(100)

**Figure 2:**
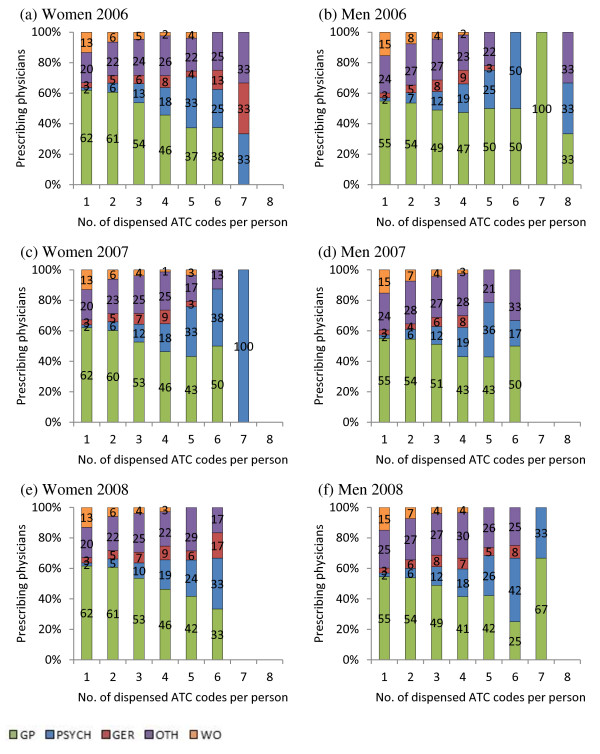
Percentage of physicians with specialist education in geriatrics (GER), psychiatry (PSYCH), general practitioners (GP), other specialists (OTH) or physicians without specialist education (WO) prescribing one or more ATC codes, level 5 (chemical substance) for mental disorders to women (a, c, e) and men (b, d, f) during the years 2006, 2007 and 2008.

GPs prescribed drugs for mental disorders to both women and men to a high extent during all three years (see Figure 2). In 2006 the prescriptions written by geriatricians to women increased with increasing number of substances, while their prescribing to men was consistently low. The prescriptions written by psychiatrists to both men and women increased with increasing number of substances.

In 2007, both men and women were prescribed drugs from geriatricians and from physicians with no specialist education to a low extent. The prescriptions written by psychiatrists to women increased with increasing number of substances, while their prescribing to men decreased with more than five substances. In the year 2008, prescriptions by psychiatrists increased with increasing number of substances for both men and women.

## Discussion

To our knowledge no other studies have evaluated the prescription of drugs for older persons with mental disorders other than dementia disorders, with a particular focus on the medical specialty of the prescribing physician. This study shows that GPs represented the main prescribers, whereas geriatricians and psychiatrists rarely prescribed drugs to older persons.

The study includes all older persons (≥65) in Sweden who received drugs dispensed at national pharmacies and thus includes the vast majority of the older population. The prevalence of prescriptions for drugs for mental disorders corresponds to approximately 7% of the older population in Sweden [4]. In fact, each year, more than half a million such prescriptions are dispensed to the older population, and the most commonly prescribed drugs are anxiolytics (benzodiazepines: oxazepam and diazepam) and tricyclic antidepressants (amitriptyline). Although previous studies on potentially inappropriate drugs for older persons suggested that benzodiazepines and tricyclic antidepressants are associated with a high risk of adverse effects and should be replaced with safer alternative drugs or therapies [30,31], the present study indicates that the use of benzodiazepines and tricyclic antidepressants was frequent. Approximately 60% of the older population was prescribed benzodiazepines, and only a minor decrease was observed over the course of the study. Furthermore, antidepressants such as mirtazapine are shown to involve high risk for adverse effects, including stroke or attempted suicide [32], and despite its association with adverse effects in older persons, prescriptions for mirtazapine tended to increase over the years. These findings highlight the clinical relevance of thoroughly and regularly evaluating the continuous treatment of older persons.

Whereas approximately 85% of the older population received only one chemical substance intended for treating mental disorders, approximately 15% received two or more substances. Although this finding per se does not indicate polypharmacy or risk for adverse drug reactions, previous studies of the use of multiple drugs in older persons suggested that it may result in inferior self-rated health [33] and increased depressive symptoms [34].

The importance of expertise in geriatrics, gerontology and psychiatry when treating older persons in general and those with mental disorders in particular has been debated [35-37]. Physicians without specialist education prescribed all types of psychotropics, across all age groups. As licensed physicians without specialist education in Sweden have undergone at most two weeks of training and education in geriatrics [14], their prescribing behavior may be detrimental to the quality and adequacy of the treatment provided to older persons with mental disorders. The present study does, however, conclude that the majority of prescriptions for drugs for mental disorders were given by GPs, perhaps not surprising as GPs represent the majority of physicians in Sweden. GPs are recognized as an important part of psychiatric care [38]. In the present study, the tendency of GPs to prescribe drugs for mental disorders increased with the age of the patient. This corresponds to a previous study showing that although GPs are aware of the advantages of reducing the prescription of psychotropic drugs, they struggle to convince their older patients of the benefits of this [39].

A previous study has shown that licensed physicians, other than psychiatrists, inaccurately diagnose mental disorders in older persons, and consequently prescribe inappropriate drugs [40]. Improved knowledge of geriatric psychiatry, including alternative treatments for older persons with mental disorders, would likely lead to the decrease in inappropriate prescription of drugs. However, although previous research has provided strong evidence on how to improve the care of older persons with mental disorders, translation into practice is rare [41,42]. Older persons with mental disorders are consequently subjected to lack of evidence-based care. To facilitate the translation of research into practice and to meet the need for specialists in geriatric psychiatry, it is essential to launch new extended educational programs for geriatricians and psychiatrists, as well as geriatric psychiatrists [43]. At present, only 60% trained geriatricians in Sweden continue to work with older persons throughout their careers [14]; low numbers of geriatricians indicates that most older persons are treated by other specialists and could explain the relatively low percentage of drugs prescribed by geriatricians. The proportion of prescriptions from geriatricians is overall low, but does increase up to 10% with increasing age of the patients; this may reflect the tendency of older persons to be treated by geriatricians as they approach 100 years of age. The proportion of prescriptions written by psychiatrists is also remarkably low: only 5% of the older persons aged 65 to 69 received prescriptions from psychiatrists and this percentage decreased further with increasing age.

The present study shows that women who are dispensed several substances tend to receive their prescriptions from geriatricians and psychiatrists to a higher extent than men. It has been demonstrated that women in old age are more likely to be affected by mental disorders [44] and receive mental health care than men [45]; this may have increased the awareness of mental health issues among older women and led to more frequent referrals of women with mental disorders to specialists such as geriatricians and psychiatrists. This supports the necessity of continuing the discussion on gender differences in old age psychiatry and it becomes evident that new and improved approaches in the care of older persons are of utmost importance for providing the older population with adequate care and treatment.

Given the complexity and challenges entailed in providing care for older persons, an increase in the number of geriatric specialists with improved competence in psychiatry or the involvement of teams of different specialists may positively contribute to improving the quality of care. Communication between GPs and other specialists is vital for improving the care and treatment of older persons in general [46] as well as older persons with mental disorders [47,48]. In order to assist healthcare personnel in providing the highest quality care to older persons with mental disorders, it is necessary to provide appropriate and effective drug treatment.

### Strengths and limitations

The SPDR includes all prescriptions dispensed at national pharmacies and consequently includes the vast majority of the population in Sweden. The study population therefore consisted of approximately 200 000 older persons, which must be considered as a strength of this study. Nonetheless, this study had some limitations. Drugs that could be used for dementia disorders have been excluded which may have led to some types of selection bias. Due to the lack of diagnoses in the register, drug use was used as a proxy for diagnoses. Consequently, it is likely that some of the drugs that were excluded due to plausible use for dementia disorders may have been prescribed for an actual diagnosis and treatment for a mental disorder and should have been included in the study (diagnosis bias). The lack of diagnoses is a crucial limitation that may have decreased the validity of the results. To counteract this bias as far as possible, the treatment guidelines and recommendations set for each year of the study were thoroughly scrutinized and each disorder and ATC code was evaluated with the help of practicing psychiatrists in order to minimize inaccuracy. Drugs recommended as firsthand options for treating dementia disorders or symptoms during the years of the study were excluded to facilitate studying mental disorders besides dementia disorders. Consequently, this brought an unfortunate exclusion of SSRIs and antipsychotics. In addition, with the register lacking diagnoses the included drugs may have been prescribed on indications other than mental disorders; tricyclic antidepressants may be prescribed for e.g. neuropathic pain rather than depression. However, as this study aimed to present what medical specialties the prescribers hold rather than the prevalence of certain disorders, each disorder and connected drugs, including all antidepressants and antipsychotics, will be further analyzed in future studies.

With the character of the national register generalizations to older populations outside Sweden must be made with consideration. One important limitation is that the register does not include diagnoses. As a result of this limitation the interpretation of the results with respect to diagnoses and disorders may affect the outcome as the differences in prescribing rates by different specialists could be altered if diagnoses were to be included. However, the register does deliver reliable information about the competences of the prescribing physicians. Questions regarding alternative therapies and non-pharmacological treatment were not within the scope of this study. However, it would be of interest to study the frequency of prescriptions for non-pharmacological treatments by the various specialist categories. In addition, whether or not the prescribing rates by different specialists differ depending on the characteristics of the drug remain unclear and consequently future studies will include studying the prescribing rates when including e.g. SSRIs in the analyses. Possibly the prescribing differences between specialists and physicians without specialist education depend on specialists recurring more frequently to other classes of drugs different from those used by physicians without specialist education. Hence future studies may answer questions as to whether or not psychiatrists and/or geriatricians prescribe alternative treatments to a greater extent than other specialists or, alternatively, if the degree of patient contact with these specialists is overall low. In future studies we will also examine subsamples of the population and their prescriptions will be further scrutinized with respect to daily doses and comparisons to the general recommendations in order to further elucidate the subject.

## Conclusions

The majority of prescriptions of drugs for mental disorders were made by GPs and not by specialists on disorders affecting the older population (geriatricians) or specialists on mental disorders (psychiatrists). The most commonly prescribed drugs were the benzodiazepines oxazepam and diazepam, as well as the tricyclic antidepressant amitriptyline, despite the fact that clinical guidelines do not recommend these medications for regular use in older persons. Approximately 15% of the older population receiving drugs for mental disorders received several substances. The results highlight the importance of regularly evaluating pharmaceutical prescriptions to older persons and raise questions about whether the involvement of more geriatric or psychiatric specialists would improve overall care.

## Competing interests

The authors declare that they have no competing interests.

## Authors’ contributions

GM participated in the study design, data collection, data analysis, interpretation of the results, and drafting of the manuscript. IF and LWG participated in the study design, interpretation of the results and drafting of the manuscript. CL participated in the design of the study, data collection, data analysis, interpretation of the results and drafting of the manuscript. All authors read and approved the final manuscript.

## Pre-publication history

The pre-publication history for this paper can be accessed here:

http://www.biomedcentral.com/1471-244X/12/197/prepub
